# TELEPHONE FRAUD AGAINST OLDER ADULTS IN CHINA: EVIDENCE FROM THE CHINA HEALTH AND RETIREMENT LONGITUDINAL STUDY

**DOI:** 10.1093/geroni/igac059.049

**Published:** 2022-12-20

**Authors:** Rong-Fang Zhan, Elias Mpofu, Cheng Yin

**Affiliations:** University of North Texas, Corinth, Texas, United States; University of North Texas, Denton, Texas, United States; University of North Texas, Corinth, Texas, United States

## Abstract

**Background:**

Falling victim to telephone fraud is a devastating crisis for older adults because they can lose their life savings, adversely affecting their well-being. This study aimed to identify risk factors associated with telephone fraud among Chinese older adults. Method: A case-control study was conducted using the fourth wave of the 2018 China Health and Retirement Longitudinal Study data on older adults 60 years and above (N = 445 with telephone fraud experience; 9,994 with no fraud and other fraud experience). Control variables for the logistic regression analysis included the individual’s demographics of gender, residential location, marital status, and educational level.

**Results:**

Older adult males (OR = 1.424, 95% CI = 1.156-1.755, p = 0.001), urban residence (OR = 2.257, 95% CI = 1.706-2.986, p < 0.001), urban-rural integration zone (OR = 2.322, 95% CI = 1.654-3.260, p < 0.001), received higher education [OR = 1.704, 95% CI = 1.211-2.397, p = 0.007]), and higher pension classification (OR = 1.352, 95% CI = 1.220-1.500, p < 0.001) were associated with increased risks of telephone fraud.

**Conclusion:**

Telephone fraud affects older adults in China at the upper rather than lower end of the socio-economic gradient, suggesting vulnerability from their use of digital technologies compared to those on the lower ladder of the socio-economic gradient. Findings highlight the need for fraud education of older adults with access to the digital broad band to reduce their risk for telephone fraud.

